# “Well it is for their sake we are here”: meaningful work tasks from care workers’ view

**DOI:** 10.1108/WWOP-09-2017-0024

**Published:** 2018-06-11

**Authors:** Åsa Vidman, Annika Strömberg

**Affiliations:** Department of Social Work and Psychology, Faculty of Health and Occupational Studies, University of Gävle, Gävle, Sweden

**Keywords:** Qualitative, Sustainability, Meaning, Care worker, Residential care, Social care practice

## Abstract

**Purpose:**

Employees in elderly care have a high rate of sick leave. One explanation is that employees that experience a low level of meaning of work are at a higher risk for long-term sick leave. The paper aims to discuss these issues.

**Design/methodology/approach:**

This qualitative interview study aims to examine what employees in residential care facilities experience as the meaningful aspects of their work tasks. Interviews with 14 persons employed in residential care facilities were conducted.

**Findings:**

The findings show that meaningful work tasks are about organizing the work to make use of the creativity and knowledge of the staff in order to support relations with older people.

**Originality/value:**

The knowledge about what constitutes a healthy work environment is not as comprehensive as it is about what constitutes health risks. Furthermore, these issues have been considered by only a few qualitative studies about social care in the field of sick leave. Therefore, this qualitative interview study examines what employees in residential care facilities experience as meaningful aspects of their work tasks.

## Introduction

The world’s population of 80 years and older will increase from 125 to 434 million people between 2015 and 2050 (WHO, 2015). Even if many older people experience a healthy aging, the ones that require support and care are increasing (WHO, 2016). To recruit and retain staff in social care is therefore an important international issue (Chester *et al.*, 2014; Stone, 2016; Wong *et al.*, 2014).

People working directly with the social care of older people and the disabled constitute one of the largest groups of employees in Sweden (Statistic Sweden, 2015), and employees in health and social care are an occupational group with one of the highest rates of days on sick leave per employee (Swedish Social Insurance Agency, 2012). The high number of employees and the rate of sick leave in the sector highlight the need for increased knowledge about these work environments.

Among employees in health and social care, the most common reason for sick leave is problems with the musculoskeletal system, but the rate of psychiatric diagnoses, such as depression, reactions to severe stress, anxiety, or sleeping disorders, are almost as high (Swedish Social Insurance Agency, 2011). Clausen *et al.* (2012) found that in Danish elderly care organizational aspects such as leadership, employee’s influence, role conflicts, the climate in the team, and emotional demands are related to risk for long-term sick leave. Furthermore, emotional exhaustion, an aspect of burnout for employees in nursing homes and assisted living facilities, can be caused by high workload, high stress, and role conflicts (Rai, 2010).

Job burnout and emotional exhaustion can also be a reason for leaving employment (Butler *et al.*, 2010), and low psychological wellbeing predicts turnover among eldercare workers (Giver *et al.*, 2010). In addition, it has been established that job satisfaction is related to sick leave and increased risk for turnover (Josephson *et al.*, 2008; Roelen *et al.*, 2008). Factors related to the work environment are related to both job satisfaction and turnover (Gleason-Wynn and Mindel, 1999). One aspect of job satisfaction is the experience of meaningful work (Ardichivili and Kuchinke, 2009; Clausen and Borg, 2011). Employees that experience a low level of meaning of work are at a higher risk for long-term sick leave (Clausen *et al.*, 2010).

The concept “meaning of work” can be defined and understood in many ways (Noon and Blyton, 2007; Rosso *et al.*, 2010). The construction of meaning can emanate in norms and shared perceptions in a society or in an individual’s own perceptions or in both (Pratt and Ashforth, 2003). In a literature review, Rosso *et al.* (2010) identify four influences on “the meaning of work”: the self (values, motivation, and beliefs); others (coworkers, leaders, groups and communities, and family); the work context (design of job tasks, organizational mission, financial circumstances, non-work domains, and national culture); and spiritual life (spirituality and sacred callings). Wrzesniewski (2003) concluded that the way employees think about their work tasks is a central component in the “meaning of work.”

The knowledge about what constitutes a healthy work environment is not as comprehensive as it is about what constitutes health risks. Furthermore, these issues have been considered by only a few qualitative studies about social care in the field of sick leave. Therefore, this qualitative interview study examines what employees in residential care facilities experience as meaningful aspects of their work tasks.

## Methods

The empirical basis for our discussion and analysis are qualitative interviews generated in a larger project aimed to answer what a healthy work environment looks like for social care workers. Therefore, interviews have been conducted with employees in residential facilities that care for older people. We contacted managers from different-sized facilities and in different modes of operation. In two facilities, the manager did not think the facility could participate because the facility was implementing a new organization. In two elderly homes, the manager did not explain why their facilities would not participate. However, managers helped us contact staff in five residential care facilities, of which three were operated by a municipality, one by a joint-stock company, and one by a cooperative. The facilities that participated had units for persons with dementia, for somatic disorders, and for short-stay housing. The number of apartments was between 9 and 120. We instructed the managers that we wanted to meet employees that had not been on sick leave for more than two weeks the previous year.

The interviewees were 13 women and one man (31-65 years old). All except two of the interviewees were born in Sweden. Only one participant had higher education; the remaining had an upper secondary-level education. They were informed both orally and in writing about the aims of the study and that their confidentiality was guaranteed. They were also informed that their participation was voluntary and that participation would not affect their employment. When referring to an interviewee in the text, the name is fictitious.

Both of the authors participated in the interviewing. All interviews took place in a room in the facilities where the interviewees worked. They were conducted individually except for in one elderly home, where the manager asked three participants to be interviewed at the same time. Each interview started with a question such as “Can you please tell us how it comes that you are working here?” and the interviews were open-ended so as to encourage dialogue. The interviews were recorded and transcribed verbatim by the first author. The second author listened to the interviews and checked the content in the transcriptions.

The analysis was conducted by both authors. A preliminary analysis showed that one important aspect is meaningful work tasks, so in the next step we coded the interviews for tasks that the staff considered meaningful. We discussed the codes made by each of us, and the codes were organized in categories that changed until all codes were represented in a category. These processes reduced the text to codes, and after that we expanded it to a new understanding through categories, a process inspired by Coffey and Atkinson (1996) and Strauss and Corbin (1998). Data were read again to check whether the created categories reflected the way the employees expressed their experiences. Finally, we related the categories to each other.

## Findings and discussion

From the interviews, six categories were derived to reflect the way the employees talked about work tasks as healthy and meaningful: doing them well, being important, getting something back, challenge, variability, and participation and influence.

### Doing them well

Already in the first reading of the transcripts, we found it obvious that employees’ wellbeing is connected with their possibility to make life better for older people. It is a double connection: if they think they can make life better for older people, the staff feel good and if the staff are satisfied, older people are satisfied as well. For example, Anna and Britta express it like this:

Well, it is for their sake we are here. And I say that they feel well if we feel well, so it is

(Anna),

Well, that you can help someone who needs help. Somehow trying to make it as good as possible so that they are having a good life. Well, after their conditions

(Britta).

Mostly, however, they did not express this sentiment that clearly. Instead, they talked about it implicitly when explaining what they thought about a healthy working environment. Concrete examples of the work are related to activities in daily life like feeding-situations, taking a walk, sitting in the sun, painting a resident’s nails, taking a footbath, teeth-brushing, and feeding the birds, and so on. Earlier research has shown that meeting both social and emotional needs of residents are important to staff (Eldh *et al.*, 2016; Liu and Bern-Klug, 2013). In addition, staff believe quality of the care is important (Häggström *et al.*, 2004; Rakovski and Price-Glynn, 2010); if this belief is not present, staff have a tendency to turnover (Castle *et al.*, 2007). Denise and Caroline, who worked in a staff cooperative with nine residents, were very proud of the group housings’ reputation and both of them talked about the necessity of caring about residents as well as staff, next of kin, and visitors:

Well, it is because of that we are here. It is for them [the residents]

(Denise).

And when you are feeding them that it isn’t like a machine, just putting it in like this, oh no. We are careful with those things

(Caroline).

However, they are not alone with respect to feeling proud and satisfied over the opportunity of giving good care. This view is important for most of the interviewees. For example Elisabeth talked about it like this:

Many from outside say we are spoiling our pensioners, but I say you cannot spoil pensioners. But I don’t know if we maybe give too much, well, I don’t think you can. All the time you want to give and we want them to have it as normal as they can

(Elisabeth).

Elisabeth conducts a line of argument with herself. She asks whether it is possible that they are too caring with older people living in their unit. In the end, her conclusion is that they are not, as she and her workmates just try to give older people as normal a life as possible. She states that it is something desired by the staff, and she also desires to give. Therefore, a residential care facility that is organized to facilitate the ability of the staff to care and doing older people well contributes to a sense of meaningful work task for the staff.

### Being important

The category “being important” has similarities with the category “doing them well” in the sense that doing older people well can lead to a sense of being important. The category “being important,” however, consists of expressions more directly connected to a sense of being required and needed. It can be such as being asked for when having some days off or when residents want to be together with an employee. One of them simply says:

And also you feel that you are needed here of older people

(Fanny).

Just before Fanny made the above comment, we had talked about going to work despite pain, and we asked if it was a sense of duty that made her go to work. According to Friedman *et al.* (1999), a close relationship with older people increases job satisfaction. Furthermore, when asking what it is that makes Gabrielle come to work, she answered that she wanted to feel needed. Later in the interview, Gabrielle provided more details: she talked about occasions when she had arranged some amusing activities such as a Christmas meal for residents and their next of kin. She also described how she arranged a lunch for the staff. Apropos the Christmas arrangements she said as follows:

Just doing the little extra and enjoying oneself when you are going home and feel that you have done something. So that is it also a bit egoistic; you want to feel needed and hear that you have done something [important]

(Gabrielle).

Our interpretation is that she talks about that she wants to be appreciated and to be seen as irreplaceable, making a difference to others. She is aware that it is not solely her altruistic desires that move her to care for others; she enjoys caring for others and this work satisfies her own need to be appreciated and acknowledged.

### Getting something back

Just as the two previous categories have similarities, the category “getting something back” is similar to those categories. “Being important” could be an aspect of “getting something back,” and likewise “getting something back” often is sensed when “doing them well.” At any rate, this category more explicitly addresses the importance of getting something back in caring work:

It is cozy in some way with the pensioners, to sit and talk with them

(Harriet).

I can go to work just to get a smile from a pensioner (laughing). I think it is wonderful

(Elisabeth).

Also in the study by Häggström *et al.* (2004), care workers talk about a smile or a hug as rewarding. In a study by Eldh *et al.* (2016), feedback from residents is considered to be of central importance. As in the quotation above, it is common that the interviewees talk about the satisfaction of talking with older people. For example they think it is amusing and interesting to listen to stories about the residents’ lives. Listening to the residents recall memories and talking to them about their experiences gives a sense of solidarity, which can be a source of satisfaction both for older people and the staff.

### Challenge

Likewise as in other studies (Clausen and Borg, 2011; Eldh *et al.*, 2016; Podsakoff *et al.*, 2007), demands of care work are seen as challenges connected to meaning at work. When the interviewees talk about work as challenging, they mostly do it in relation to older people. Mostly it is the staff on dementia units who talk about challenge as an aspect of a satisfying work. The challenge they are talking about is to find ways to meet older people and reduce their frustration; it is a bit of a detective work:

They are getting angry and sad and [we are] trying to solve that. […] One day that lady can be the kindest in the world and you can sit and hug her and the next day she is laying and kicking and somehow how are you going to solve it?

(Ingrid).

Alleviating frustration for a person with dementia is described as a challenge. The staff expressed the need to have knowledge about both the specific person as well as dementia, and they are aware of the fact that there is a reason for a resident’s frustration. The challenge is to find out why the residents are frustrated and to help them deal with their frustration. Helping alleviate the residents’ frustration is seen as a satisfying aspect of work:

It is seldom they are sore and whining, and then it always originates in something. They are there somewhere so to speak; you’ll find them

(Elisabeth).

But it is not solely in dementia units the work is challenging; it is in short-stay housing as well. In this case, it is more a matter of meeting new people with different needs and personalities and to develop new skills:

I think working with short-stay living residents is rewarding. It changes all the time; it does not go in the same wheel track, but it changes a bit. Then there are new challenges. I think it is rewarding

(Jan).

The challenge in short-stay units is therefore in a way about variability, but in our analysis, it is not just about that. Instead, the staff want to be challenged and feel the satisfaction of solving problems.

### Variability

The category “variability” consists of different experiences. One aspect is that it can be satisfying to meet many people during the day:

Before you were working in the whole house. You were more staff; you met them more often. Nowadays, it is not often you see anyone from another unit

(Gabrielle).

The residential care facilities where all the interviewees were working were divided into small units with at most ten rooms or apartments for residents. In addition, the staff are organized so that they mostly work solely at that unit. According to Pekkarinen *et al.* (2004), the risk for work stress increases the larger the unit, but the quotation above shows that Gabrielle is not quite pleased with that organization. Even if the interviewees like to have variability in their work, overall they seem to like to work in small units.

The interviewees who work at units for short-stay housing experience higher resident turnover and different problems to deal with very stimulating. They think they are involved in many different tasks, often novel tasks, so they are always learning. It has been shown that a variety in work increases job satisfaction (Friedman *et al.*, 1999). Another way of expressing the category “variability” is experiencing an unexpected incident as a positive situation. If you feel safe with the organization and the routines, the unexpected can counteract boredom:

I like working with people. And it is always different. Still there are some routines; it is so, and you have to keep some kind [of order]. But what I mean is that you never know how the day ends

(Ingrid).

Variability is also about new routines and ways of doing the work. It makes the work more enjoyable and gives the staff a sense that they are constantly developing their professional skills. To work at a unit where the staff are willing to try innovations can be something that give satisfaction, but not everyone agrees:

There can be someone who “No, I won’t, I am not ready for that change.” But here all of us have a very constructive attitude. We want new things to happen all the time. No one is stuck or resists when we are going to do something

(Kristina).

Furthermore, the category “variability” includes the aspect that most of the interviewees have special tasks or commissions that give them opportunity to have a variety in their work. These tasks include duties concerning schedules, planning the work, IT, food, activities, development, co-ordination or commissions of trust like being a union or safety representative. This kind of work provides the staff variation in their work tasks:

Well, I am also doing a lot more work than care work so that I can get away from here sometimes

(Lena).

Although these special tasks can be seen as a relief from the ordinary work, it is also rewarding as it gives the staff the opportunity to have influence in their work and the possibility to develop new skills. To learn about different kinds of needs and ways to satisfy these needs is about professional development.

### Participation and influence

Employees feel empowered when they have an opportunity to positively influence their work environment. This influence can be in the everyday work as well as in overall planning. In addition, the ability to decide one’s own work as well as being involved in decision-making can make work more pleasant. Influence is described as a satisfying part of the work for the staff. For example, Anna is very satisfied with the different commissions she have been entrusted. One such commission is to be a leader of development, and she says:

And I am also coordinator in our unit. Then you manage some paper and things like that and handle information from the manager and from the house and what’s happening. […] Thus we are supposed to come up with ideas. Absolutely. We are having a dialogue with the managers in the housing. Like now we have been taken care of the days for planning the work. Then we have had to sort out by ourselves how to plan activities during a morning

(Anna).

The two excerpts above illustrate that Anna is involved in different processes that involve influence. Jenkins and Allen (1998) found that the opportunity to participate in work-related decisions among residential workers decreased negative interaction with the residents, although Brannon *et al.* (2002) did not find a significant relationship between involvement in care planning and turnover. However, high levels of influence decrease the risk for long-term sick absence (Clausen *et al.*, 2012). Emamgholizadeh *et al.* (2011) concluded that participation in decision-making is significantly related to meaning, which in their study was seen as an aspect of empowerment. Empowerment, in turn, decreases the risk for burnout (Hochwälder, 2007). According to Oxenstierna *et al.* (2005), low employee participation in decision-making correlates with worse employee health.

A meaningful and satisfying work environment can be understood by defining what it is not. Denise, for example, talked about why she left a previous residential care facility:

We who were employed had our backpacks with us. We came from different places, but no one asked us “What do you think?” or “You who have experience” but these [owners] did not have any experience [of dementia care], so from the beginning they sort of did not listen nor did they want any help from us

(Denise).

To have experience and knowledge that is not demanded reduces the sense of being important and skilled and it negatively influences job satisfaction. Having respect for one’s suggestions increases job satisfaction (Friedman *et al.*, 1999). Opportunity to be a part of the planning process increases the sense of meaning in the work, which is what Anna is talking about in the following excerpt work:

We did a lot of schedules before; it is also a task I have been doing, and I have been doing it here as well. And that is also a way of participating in decisions and having influence in the working hours

(Anna).

## Concluding discussion

**Figure 1:**
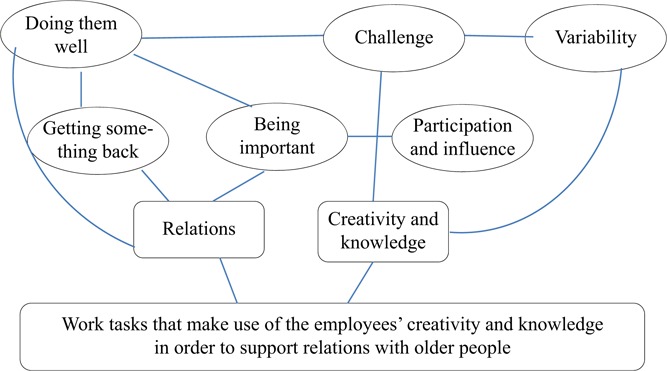
Aspects of work tasks employees experience as healthy and meaningful

All together the findings in this study suggest that employees in elderly care facilities see their work tasks as meaningful if their facilities are flexible and respect the needs of both older people and the staff. In the analysis of the interviews, we have highlighted six categories that describe meaningful work tasks. These categories are linked and are not single and separate aspects of meaningful work tasks. Figure 1 shows six categories that together constitute two themes that can be related to the main theme of the study.

As mentioned before, “doing them well” has many similarities to a sense of “being important” even if a sense of importance is not always connected to the work directly with older people. Both those categories contribute to the category “getting something back.” “Being important” can also be both a result of and lead to “creativity and development.” “Variability” in work can be a challenge, and work where employees can use their creativity and knowledge is both required and a consequence of challenging as well as variable work tasks. Work with possibility to participate and to influence strengthens the sense of being important. This type of work also gives opportunities to use one’s creativity and knowledge and vice versa. Establishing a good relationship with older people requires creativity and knowledge.

Although these six categories are linked, they are different in at least two ways: some of the categories address informal work connected to everyday life and relationships with older people and some categories address formal and structural parts of work. The staff want to be trusted with the responsibility to make decisions about everyday life and to participate in decisions of a more formal kind. They want to take responsibility over having good relations with older people and over gaining new skills, but most of all they want the working life to be organized in a way that supports relationships and professional development in ways that they can use their creativity, knowledge, and skills.

What characterize meaningful work tasks differs according to work context. Therefore, the findings in this study are influenced by the specific characteristic of older people care sector. Hence the study of the manufacture sector, for example, may produce different results. Therefore it is not remarkable that the findings in this study support the idea that the staff saw work tasks related to the relationship between staff and older people and their wellbeing as important. The contribution of this study is rather that this part of the care work should not be considered the complete picture of what constitutes meaningful work tasks. One important part is about structure and employees’ possibility to influence and make use of their competence and creativity. Variability in work tasks (often tasks not directly related to work with older people) is also important. The study indicates that meaning is created in the dialectic between the soft, relational aspects of the care work and the more structural parts of the work. This observation is shown by the findings that the six categories of meaningful work tasks are interwoven, overlapping, and related to each other. Therefore, it does not seem to be fruitful to try to create meaning using separate solutions. Instead, we emphasize a focus on process, as meaningful work is about organizing work in a way that gives employees an opportunity to use their competence and creativity, meet older people and their needs by a relational way of working.

The study presented here contributes by focusing on the healthy aspects that employees highlight as important for a healthy work environment. Focus on the healthy parts of the work environment gives the possibility to work in a more preventive way rather than taking care of already existing problems, that is, the results of this study have implications on leadership and management of elderly care. Leadership and management set the structures and frames for organizing daily work, so the discussions and planning about healthy aspects of meaningful work tasks have to be held on a strategic level. There is a need for further discussions about how to support and manage a healthy work environment.

## Methodological considerations

The findings presented in this paper are a part of a larger project aimed to illuminate what employees in elderly care consider a healthy work environment. One aspect the employees emphasized in the project was meaningful work tasks. Therefore, we focused on how the employees experience meaningful work tasks. Although the data did not have the point of departure in meaningful work tasks and the interviews did not explicitly focus on meaning, the follow-up questions and the dialogue sometimes included those aspects. Consequently, a study with an explicit purpose to focus on meaningful work tasks would perhaps give a deeper understanding. Therefore we want to emphasize the need for further research that focuses on the dialectic between relational and structural aspects of meaningful work tasks.

## References

[ref001] Ardichivili A. and Kuchinke K.P. (2009), “International perspectives on the meanings of work and working: current research and theory”, Advances in Developing Human Resources, Vol. 11 No. 2, pp. 155-67.

[ref002] Brannon D., Zinn J.S., Mor V. and Davis J. (2002), “An exploration of job, organizational, and environmental factors associated with high and low nursing assistant turnover”, The Gerontologist, Vol. 42 No. 2, pp. 159-68. 11914459 10.1093/geront/42.2.159

[ref003] Butler S.S., Simpson N., Brennan M. and Turner W. (2010), “Why do they leave? Factors associated with job termination among personal assistant workers in home care”, Journal of Gerontological Social Work, Vol. 53 No. 8, pp. 665-81. 20972925 10.1080/01634372.2010.517236

[ref004] Castle N.G., Engberg J., Anderson R. and Men A. (2007), “Job satisfaction of nurse aides in nursing homes: intent to leave and turnover”, The Gerontologist, Vol. 47 No. 2, pp. 193-204. 17440124 10.1093/geront/47.2.193

[ref037] Chester H., Hughes J. and Challis D. (2014), “Commissioning social care for older people: influencing the quality of direct care”, Ageing & Society, Vol. 34 No. 6, pp. 930-50.

[ref005] Clausen T. and Borg V. (2011), “Job demands, job resources and meaning at work”, Journal of Managerial Psychology, Vol. 26 No. 8, pp. 665-81.

[ref006] Clausen T., Christensen K.B. and Borg V. (2010), “Positive work-related states and long-term sickness absence: a study of register-based outcomes”, Scandinavian Journal of Public Health, Vol. 38 No. S3, pp. 51-8. 21172771 10.1177/1403494809352105

[ref007] Clausen T., Nielsen K., Carniero I.G. and Borg V. (2012), “Job demands, job resources and long-term sickness absence in the Danish eldercare services: a prospective analysis of register-based outcomes”, Journal of Advanced Nursing, Vol. 68 No. 1, pp. 127-36. 21658095 10.1111/j.1365-2648.2011.05724.x

[ref008] Coffey A. and Atkinson P. (1996), Making Sense of Qualitative Data: Complementary Research Strategies, SAGE Publications, Thousand Oaks, CA.

[ref009] Eldh A.C., van der Zijpp T., McMullan C., McCormack B., Seers K. and Rycroft-Malone J. (2016), “‘I have the world’s best job’ – staff experience of the advantages of caring for older people”, Scandinavian Journal of Caring Sciences, Vol. 30 No. 2, pp. 365-73. 26265314 10.1111/scs.12256

[ref010] Emamgholizadeh S., Matin H.Z. and Razavi H.R. (2011), “Is participation in decision making related to employee’s empowerment?”, African Journal of Business Management, Vol. 5 No. 9, pp. 3504-10.

[ref011] Friedman S.M., Daub C., Cresi K. and Keyser R. (1999), “A comparison of job satisfaction among nursing assistants in nursing homes and the Program of All-Inclusive Care for the Elderly (PACE)”, The Gerontologist, Vol. 39 No. 4, pp. 434-9. 10495581 10.1093/geront/39.4.434

[ref012] Giver H., Faber A., Hannerz H., Strøyer J. and Rugulies R. (2010), “Psychological well-being as a predictor of dropout among recently qualified Danish eldercare workers”, Scandinavian Journal of Public Health, Vol. 38 No. 3, pp. 239-45. 19815680 10.1177/1403494809348939

[ref013] Gleason-Wynn P. and Mindel C.H. (1999), “A proposed model for predicting job satisfaction among nursing home social workers”, Journal of Gerontological Social Work, Vol. 32 No. 3, pp. 65-79.

[ref014] Häggström E., Skovdahl K., Fläckman B., Kihlgren A.L. and Kihlgren M. (2004), “To feel betrayed and to feel that you are betraying the older residents: caregivers’ experiences at a newly opened nursing home”, Journal of Clinical Nursing, Vol. 13 No. 6, pp. 687-96. 15317508 10.1111/j.1365-2702.2004.00939.x

[ref015] Hochwälder J. (2007), “The psychosocial work environment and burnout among Swedish registered and assistant nurses: the main, mediating, and moderating role of empowerment”, Nursing and Health Sciences, Vol. 9 No. 3, pp. 205-11. 17688479 10.1111/j.1442-2018.2007.00323.x

[ref016] Jenkins H. and Allen C. (1998), “The relationship between staff burnout/distress and interactions with residents in two residential homes for older people”, International Journal of Geriatric Psychiatry, Vol. 13 No. 7, pp. 466-72. 9695036 10.1002/(sici)1099-1166(199807)13:7<466::aid-gps799>3.0.co;2-v

[ref017] Josephson M., Lindberg P., Voss M., Alfredsson L. and Vingård E. (2008), “The same factors influence job turnover and long spells of sick leave – a 3-year follow-up of Swedish nurses”, European Journal of Public Health, Vol. 18 No. 4, pp. 380-5. 18292122 10.1093/eurpub/ckn009

[ref018] Liu J. and Bern-Klug M. (2013), “Nursing home social service directors who report thriving at work”, Journal of Gerontological Social Work, Vol. 56 No. 2, pp. 127-45. 23350567 10.1080/01634372.2012.750255

[ref019] Noon M. and Blyton P. (2007), The Realities of Work: Experiencing Work and Employment in Contemporary Society, Palgrave, Basingstoke.

[ref020] Oxenstierna G., Ferrie J., Hyde M., Westerlund H. and Theorell T. (2005), “Dual source support and control at work in relation to poor health”, Scandinavian Journal of Public Health, Vol. 33 No. 6, pp. 455-63. 16332610 10.1080/14034940510006030

[ref021] Pekkarinen L., Sinervo T., Perälä M.-L. and Elovainio M. (2004), “Work stressors and the quality of life in long-term care units”, The Gerontologist, Vol. 44 No. 5, pp. 633-43. 15498839 10.1093/geront/44.5.633

[ref022] Podsakoff N.P., LePine J.A. and LePine M.A. (2007), “Differential challenge stressor-hindrance stressor relationships with job attitudes, turnover intentions, turnover, and withdrawal behavior: a meta-analysis”, Journal of Applied Psychology, Vol. 92 No. 2, pp. 438-54. 17371090 10.1037/0021-9010.92.2.438

[ref023] Pratt M.G. and Ashforth B.E. (2003), “Fostering meaningfulness in working and at work”, in Cameron K.S., Dutton J.E. and Quinn R.E. (Eds), Positive Organizational Scholarship. Foundations of a New Discipline, Berett-Kohler Publishers, San Francisco, CA, pp. 309-27.

[ref024] Rai G.S. (2010), “Burnout among long-term care staff”, Administration in Social Work, Vol. 34 No. 3, pp. 225-40.

[ref025] Rakovski C.C. and Price-Glynn K. (2010), “Caring labour, intersectionality and worker satisfaction: an analysis of the National Nursing Assistant Study”, Sociology of Health & Illness, Vol. 32 No. 3, pp. 400-14. 19891615 10.1111/j.1467-9566.2009.01204.x

[ref026] Roelen C.A.M., Koopmans P.C., Notenbomer A. and Groothoff J.W. (2008), “Job satisfaction and sickness absence: a questionnaire survey”, Occupational Medicine, Vol. 58 No. 8, pp. 567-71. 18775871 10.1093/occmed/kqn113

[ref027] Rosso B.D., Dekas K.H. and Wrzesniewski A. (2010), “On the meaning of work: a theoretical integration and review”, Research in Organizational Behavior, Vol. 30 No. 1, pp. 91-127.

[ref028] Statistic Sweden (2015), “The Swedish Occupational Register with statistics”, available at: www.scb.se/contentassets/982742930b774ff49ba5a7056d069992/am0208_2015a01_sm_am33sm1701.pdf (accessed August 28, 2017).

[ref029] Stone R.I. (2016), “The migrant direct care workforce: an international perspective”, Generations, Vol. 40 No. 1, pp. 99-105.

[ref030] Strauss A. and Corbin J. (1998), Basics of Qualitative Research: Techniques and Procedures for Developing Grounded Theory, 2nd ed., SAGE Publications, Thousand Oaks, CA.

[ref031] Swedish Social Insurance Agency (2011), “Sjukskrivningsdiagnoser i olika yrken: Startade sjukskrivningar (>14 dagar) per diagnos bland anställda i olika yrken år 2009”, Social Insurance Report No. 2011:17, available at: www.forsakringskassan.se/wps/wcm/connect/84cb4254-0889-4a51-9601-e4bc82931872/socialforsakringsrapport_2011_17.pdf?MOD=AJPERES (accessed August 28, 2017).

[ref032] Swedish Social Insurance Agency (2012), “Sjukskrivning i olika yrken under 2000-talet: Antal ersatta sjukskrivningsdagar per anställd år 2002-2010”, Social Insurance Report No. 2012:14, available at: www.forsakringskassan.se/wps/wcm/connect/53a6e173-bad8-4ee2-84df-d909665bfc13/socialforsakringsrapport_2012_14.pdf?MOD=AJPERES (accessed August 28, 2017).

[ref033] WHO (2015), “Ageing and health”, Fact sheet No. 404, available at: www.who.int/mediacentre/factsheets/fs404/en/ (accessed August 28, 2017).

[ref034] WHO (2016), “Multisectoral action for a life course approach to healthy ageing: draft global strategy and plan for action on ageing and health”, Sixty-ninth World Health Assembly, A69/17, April 22, available at: http://apps.who.int/gb/ebwha/pdf_files/WHA69/A69_17-en.pdf?ua=1 (accessed August 28, 2017).

[ref035] Wong W.N.K., Kwok S.T. and Lee T.Y.A. (2014), “Recruitment challenges facing elderly care service providers in Hong Kong”, British Journal of Healthcare Management, Vol. 20 No. 4, pp. 184-90.

[ref036] Wrzesniewski A. (2003), “Finding positive meaning in work”, in Cameron K.S., Dutton J.E. and Quinn R.E. (Eds), Positive Organizational Scholarship. Foundations of a New Discipline, Berett-Kohler Publishers, San Francisco CA, pp. 296-308.

